# Realization of ground-state artificial skyrmion lattices at room temperature

**DOI:** 10.1038/ncomms9462

**Published:** 2015-10-08

**Authors:** Dustin A. Gilbert, Brian B. Maranville, Andrew L. Balk, Brian J. Kirby, Peter Fischer, Daniel T. Pierce, John Unguris, Julie A. Borchers, Kai Liu

**Affiliations:** 1 Department of Physics, University of California, Davis, California 95616, USA; 2 NIST Center for Neutron Research, National Institute of Standards and Technology, Gaithersburg, Maryland 20899, USA; 3 Center for Nanoscale Science and Technology, National Institute of Standards and Technology, Gaithersburg, Maryland 20899, USA; 4 Maryland Nanocenter, University of Maryland, College Park, Maryland 20742, USA; 5 Center for X-Ray Optics, Lawrence Berkeley National Laboratory, Berkeley, California 94720, USA; 6 Department of Physics, University of California, Santa Cruz, California 94056, USA

## Abstract

The topological nature of magnetic skyrmions leads to extraordinary properties that provide new insights into fundamental problems of magnetism and exciting potentials for novel magnetic technologies. Prerequisite are systems exhibiting skyrmion lattices at ambient conditions, which have been elusive so far. Here, we demonstrate the realization of artificial Bloch skyrmion lattices over extended areas in their ground state at room temperature by patterning asymmetric magnetic nanodots with controlled circularity on an underlayer with perpendicular magnetic anisotropy (PMA). Polarity is controlled by a tailored magnetic field sequence and demonstrated in magnetometry measurements. The vortex structure is imprinted from the dots into the interfacial region of the underlayer via suppression of the PMA by a critical ion-irradiation step. The imprinted skyrmion lattices are identified directly with polarized neutron reflectometry and confirmed by magnetoresistance measurements. Our results demonstrate an exciting platform to explore room-temperature ground-state skyrmion lattices.

## Abstract (web summary)

Magnetic skyrmions are particle-like magnetization textures which may be manipulated in thin-film device applications. Here, the authors demonstrate the formation and control of room-temperature artificial skyrmion lattices in Co/Pd multilayers, defined by local ion irradiation and an array of magnetic vortex discs.

## Graphical abstract

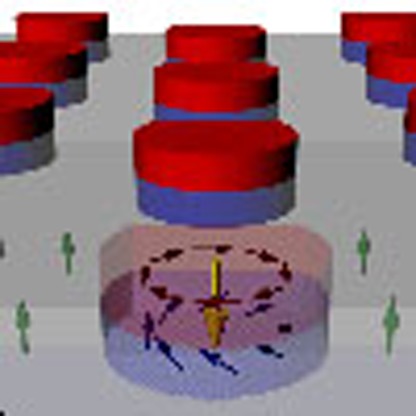

The unique spin texture in magnetic skyrmions leads to a host of fascinating phenomena due to the topologically protected quantum state^1^,^2^,^3^,^4^,^5^,^6^ and emergent electromagnetic field^7^,^8^,^9^, offering great potential for novel concepts in low-dissipation magnetic information storage or skyrmionics^5^,^7^,^10^,^11^,^12^. For example, the recent experimental observation that current-driven skyrmion crystal motion only requires current densities that are several orders of magnitude smaller than that necessary for typical current-driven domain wall motion, has sparked intense interest in the field^13^.

The Dzyaloshinskii–Moriya interaction is a common mechanism to generate magnetic skyrmions in certain non-centrosymmetric magnets such as MnSi(Fe,Co), (Mn,Fe)Ge, Cu_2_OSeO_3_ and La_2_Cu_0.97_Li_0.03_O_4_, which are usually limited to low temperatures and in finite magnetic fields^3^,^6^,^7^,^14^,^15^,^16^,^17^. Artificial structures such as thin films have been shown to extend the skyrmion phase space^18^. Frustrated exchange interactions^19^ and four-spin exchange interactions^4^ have also been shown to lead to atomic-sized skyrmions at low temperatures. Prerequisite, however, for systematic studies of the unique properties and the technological exploitation of magnetic skyrmions is a ground state, which establishes itself at ambient conditions.

In systems with perpendicular magnetic anisotropy (PMA), the competition between PMA and dipolar interactions results in the formation of magnetic bubbles^20^,^21^, some of which are actually magnetic skyrmions with topological characteristics^22^. In conjunction with interfacial Dzyaloshinskii–Moriya interaction, breaking the mirror symmetry^4^,^11^,^23^,^24^, such PMA systems show potential for room temperature control of skyrmions. However, as the formation and stabilization of bubbles in PMA systems generally require the application of an external magnetic field, the subsequent skyrmion state is not the ground state in zero field.

Recent theoretical work has suggested that skyrmions may be artificially created through nanopatterning^25^; regular arrays of skyrmions then form a skyrmion lattice (SL). By fabricating vortex-state nanodots^26^ on a PMA film, the exchange interaction is proposed to imprint a Bloch SL into the underlying film^27^: the region underneath the dots have in-plane winding of the magnetization (known as chirality or circularity, with the former preserving a handedness)^28^,^29^, coupled with out-of-plane (OOP) magnetization in the middle (polarity of the core) and at outer boundaries of the in-plane magnetization, but in opposite directions. The integrated solid angles spanned by the moments in these skyrmions are ±4π, yielding a skyrmion number^7^ of 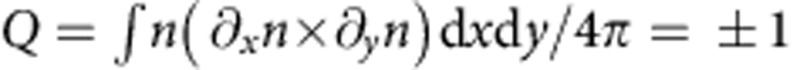, where *n*(*x*,*y*) is the normalized magnetization field at location (*x*,*y*). These artificial SLs are expected to be stable and accessible over wide temperature and field ranges and in the absence of the Dzyaloshinskii–Moriya interaction.

However, there are key open challenges in using such artificial SLs towards room-temperature skyrmionics: imprinting the vortex configuration from the patterned dots into the PMA underlayer, and polarity and circularity control. At the interface between the dot and the underlayer, the PMA will be in direct competition with the magnetostatic interaction, which differentiates an SL versus a vortex lattice (VL) on top of a PMA underlayer. Although earlier studies on artificial skyrmions have shown encouraging results^30^,^31^, so far there has been no report of direct evidence of the imprinted skyrmions. In addition, controlling vortex core polarity in the hybrid structure over the entire array to be (anti-)parallel to the PMA underlayer magnetization is critical in setting the skyrmion number *Q* to be 0 or ±1. Finally, controlling the in-plane vortex circulation direction of all dots in the lattice is important. In individual skyrmions, the opposite chiralities of the in-plane vortex texture are topologically equivalent^30^; however, in extended SLs, circulation control may affect skyrmion interactions.

Here, we present direct experimental evidence of artificial SLs with a stable ground state at room temperature. Our approach is to pattern vortex-state Co nanodots in hexagonal arrays via electron-beam lithography on top of a Co/Pd thin film with PMA and prepare the skyrmion state using a specific magnetic field sequence. These hybrid structures exhibit circularity and polarity control over the entire lattice, and the vortex structure of the nanodots is imprinted into the PMA underlayer to form SLs. They offer a convenient and powerful platform to explore skyrmion physics and topological phenomena, even at room temperature and in the absence of any magnetic field.

## Results

### Experimental design

**Figure 1:**
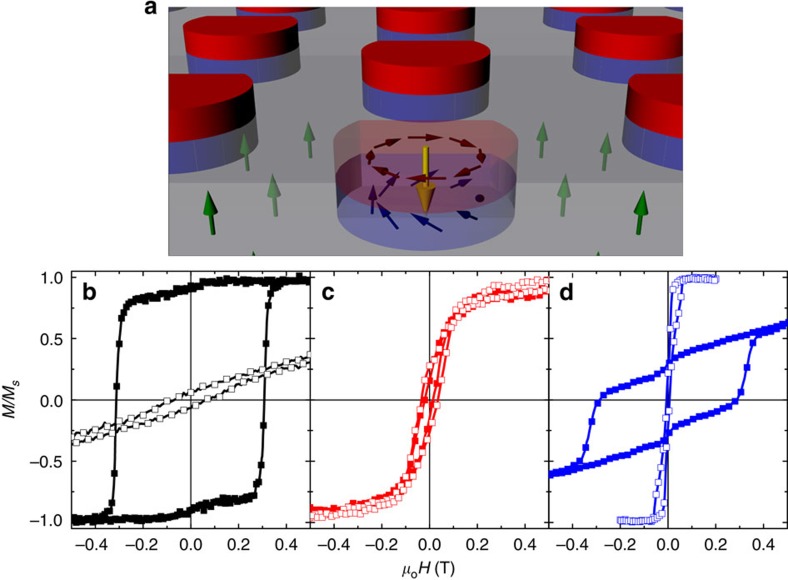
Schematic diagram of the artificial skyrmion lattices and measured magnetic hysteresis loops. (**a**) The hybrid structure consists of Co dots (red) on top of Co/Pd PMA underlayer (grey) where the in-plane spin texture of the Co dots (purple arrows) is imprinted into an irradiated Co/Pd region (light blue) underneath the dots (tilted blue arrows). Green and yellow arrows indicate the moments in the Co/Pd underlayer and the core region of the (imprinted) vortex, respectively. Major in-plane (open symbols) and perpendicular (solid symbols) hysteresis loops are shown for (**b**) the Co/Pd underlayer as grown, (**c**) the irradiated Co/Pd witness sample and (**d**) the hybrid Co+Co/Pd sample.

Hybrid structures of asymmetric Co nanodot arrays with in-plane magnetic easy axis grown on Co/Pd thin film underlayers with PMA, illustrated in Fig. 1a, were fabricated by a three-step process: a Co/Pd film was first sputter deposited, and subsequently spin-coated with a polymethyl methacrylate (PMMA) film; arrays of asymmetric edge-cut anti-dots were patterned into this polymer layer by e-beam lithography. Next, the film was irradiated with energetic Ar^+^ ions, thus modifying the multilayer structure in the exposed dot areas and tilting their easy axis in-plane^32^. Lastly, Co was sputtered over the irradiated anti-dot arrays, forming edge-cut Co dots after a lift-off process. The Co/Pd films exhibit (111) texture and the Co dots are polycrystalline, respectively, as reported in previous studies^33^. The skyrmion texture was then prepared by a designed field sequence, as detailed in Methods: the Co/Pd underlayer was first saturated in the positive perpendicular direction; then the perpendicular field was removed and a much smaller in-plane field was applied to saturate the Co dots parallel to their flat edge; lastly, a negative perpendicular field was applied as the in-plane field was removed to set the polarity, and finally the perpendicular field was removed.

Hysteresis loops for a Co/Pd thin-film reference sample before and after Ar^+^ irradiation, and those for the hybrid sample are shown in Fig. 1b–d, respectively. The as-grown Co/Pd films exhibit strong PMA with an OOP remanence of essentially unity and a coercivity of *μ*_*o*_*Η*_*C*_=320 mT (Fig. 1b). In contrast, for the Co/Pd film irradiated by Ar^+^ over the entire area, the in-plane and perpendicular hysteresis loops are nearly identical (Fig. 1c), confirming that the PMA has been successfully suppressed. The hybrid sample in the perpendicular geometry retains the characteristics of the unirradiated Co/Pd film (Fig. 1d), illustrating that the processing did not damage the perpendicular layer other than as designed; in the in-plane geometry the loop exhibits a pinched shape, typical of magnetic vortex reversal^34^, with nucleation and annihilation fields of ∼10 and 60 mT, respectively. These results demonstrate that indeed the hybrid sample has realized the designed magnetic configurations, that is, the Co/Pd underlayer is perpendicularly magnetized in a single domain state, while the Co dots are in an in-plane vortex state. After the artificial skyrmions are set by the aforementioned magnetic field sequence, they are expected to be stable over wide magnetic field and temperature (persist to near the Co/Pd Curie temperature) ranges.

To confirm the SL, we demonstrate in the following circularity and polarity control in the vortex region (Co dots), and an imprinting of the vortex structure from the dot into the underlayer.

### Circularity control

**Figure 2:**
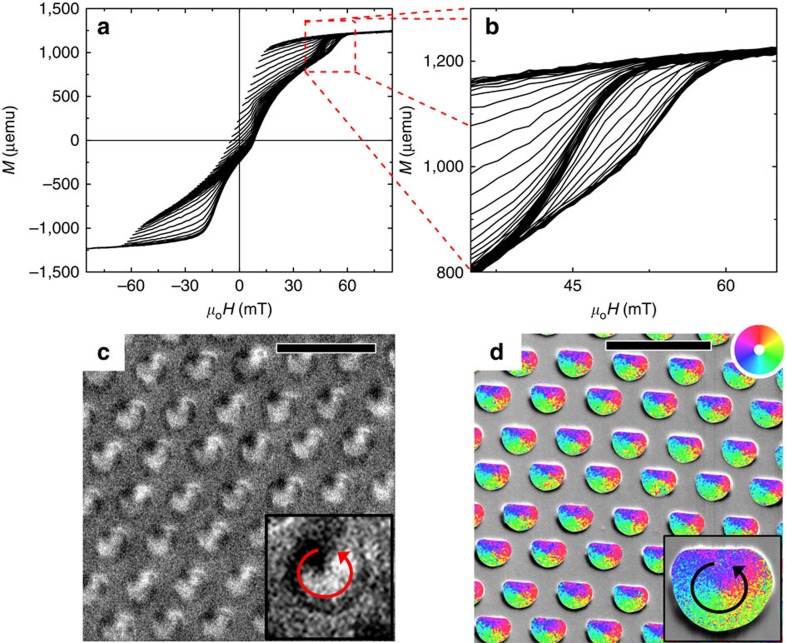
Circularity control. (**a**) Family of FORCs of the hybrid sample measured in the in-plane geometry. The zoomed-in view in (**b**) illustrates essentially two discrete vortex annihilation fields. Remanent-state (**c**) MFM and (**d**) SEMPA (superimposed onto a scanning electron microscopy image of the dots) images, after saturating the dots in an in-plane field parallel to the flat edge of the dots to the right, indicate circularity control. Scale bar, 2 μm. A key to the magnetization winding direction is shown in the insets.

Circularity control of the hybrid sample is illustrated in first-order reversal curves (FORCs)^35^,^36^,^37^ measured in the in-plane geometry, shown in Fig. 2a, where the applied field is parallel to the flat edge of the dots. Specifically, FORCs approaching positive saturation reveal essentially two discrete vortex annihilation fields, as highlighted in Fig. 2b, depending on their reversal fields. FORCs reversing near the negative saturation conform onto the major loop, delineated by the outer boundaries of the family of FORCs; in contrast, those reversing sooner, particularly with reversal fields in the first quadrant, reveal a smaller vortex annihilation field. These discrete annihilation fields are manifestations of circularity controlled vortices being annihilated from the flat versus rounded edge of the dots, as shown previously^26^,^38^. Remanent-state magnetic imaging performed with magnetic force microscopy (MFM) and scanning electron microscopy with polarization analysis (SEMPA), after aforementioned SL-setting field sequence, provide direct evidence of circularity control over the dot arrays, as shown in Fig. 2c,d, respectively. This is further confirmed by magnetic transmission X-ray microscopy (MTXM)^29^ study of similar samples (Supplementary Fig. 1; Supplementary Note 1).

### Polarity control

The large sample area (1 cm^2^) allows us to distinguish magnetometry signatures of the SL to confirm polarity control. The SL is prepared with the core polarity opposite to the underlayer magnetization by applying a perpendicular bias field as the vortex is nucleated. Similarly, by instead applying a perpendicular bias parallel to the underlayer magnetization the polarity can be aligned with the underlayer. The resultant structure is not an SL, since the phase shift of the perpendicular magnetization across this structure will be 0, representing just a VL on top of a PMA underlayer (*Q*=0 in Co/Pd). The SL and VL will have different perpendicular remanent magnetizations as a result of the vortex core orientation. In addition, the bias field can be removed entirely during the vortex nucleation in the Co dots, resulting in a random distribution of the core polarity. This mixed lattice (ML) is expected to have a remanence between the cases with ordered polarity, and a reversal behaviour indicative of both features.

**Figure 3:**
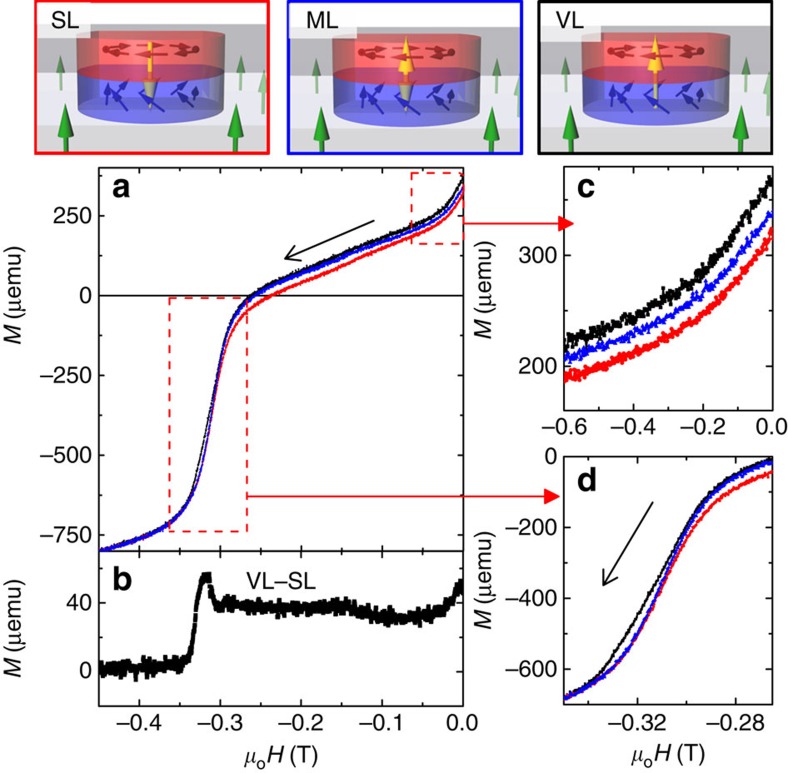
Polarity control. Top row shows schematic illustrations of the SL, VL and ML states. The yellow arrows mark the core direction in the vortex and the imprinted region, while the other arrows represent the magnetic moments in other parts of the structure. (**a**) Magnetization curves, with the field sweeping from zero to negative saturation, for the hybrid structure prepared into the SL (red), VL (black) and ML (blue) states at remanence. (**b**) The image highlights the magnetization difference between the VL and SL, and zoomed-in views of the magnetization curves in dashed boxes are shown in (**c**) near zero field and (**d**) ∼320 mT where the Co/Pd underlayer starts its reversal, respectively.

Remanent state of the hybrid structure is configured into the SL, VL and ML, respectively (procedures described in Methods). As the perpendicular field was swept from 0 to negative saturation, the magnetization curves reveal clear differences, as shown in Fig. 3a (1 μemu=1 nA m^2^), with the difference between SL and VL highlighted in Fig. 3b. In the early stage of the magnetization reversal, illustrated in Fig. 3c, the case of parallel alignment between the core and underlayer (VL) exhibits the largest magnetization, while anti-parallel alignment (SL) shows the smallest, and random alignment (ML) midway in between the VL and SL curves. The magnetization difference between the VL and SL configurations divided by the number of dots corresponds to a region with perpendicular moment of ∼30 nm radius within each dot. This value is an upper limit of the core size, since the surrounding region of the core and imprinted skyrmions also contribute to the signal. This difference remains almost constant along the field sweep until the underlayer's reversal field at ∼−0.3 T, indicating the stability of the skyrmions. As the Co/Pd underlayer starts its reversal, highlighted in Fig. 3d, the abrupt magnetization drop occurs first in the SL configuration and last in the VL. This difference arises because in the SL the oppositely oriented cores will facilitate the nucleation of reversal domains, whereas in the VL the parallel cores will not. The difference in the nucleation field for SL versus VL is indicative of the topological effect on the nucleation process of Co/Pd reversal domains. The ML configuration shows a mixed reversal, evolving from tracking the VL to the SL. This behaviour is consistent with the presence of both types of pole configurations with opposite polarities. The magnetization difference near remanence and the field sweep thus clearly demonstrates the polarity control.

### Imprinted structure

**Figure 4:**
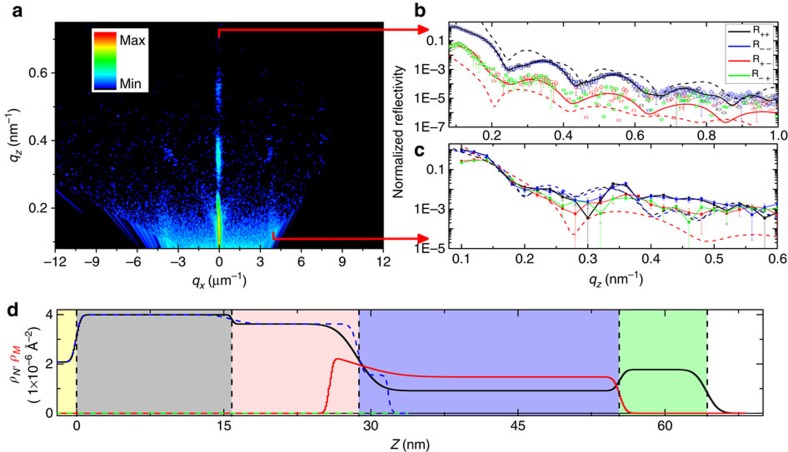
Polarized neutron reflectometry. (**a**) Position-sensitive area-detector image of the (*q*_*x*_, *q*_*z*_) unpolarized neutron scattering, with polarized traces along (**b**) the specular (*q*_*x*_=*0*) and (**c**) the first off-specular (*q*_*x*_=3.6 μm^−1^) reflections. The fitted depth-dependent nuclear (*ρ*_*Ν*_, solid black and dashed blue curves) and magnetic (*ρ*_*Μ*_, solid red and dashed green curves) scattering densities from the specular measurement are shown in (**d**), over the protected film (dashed curves) and the vortex region (solid curves). On top of the Si substrate (yellow region), at increasing depth, the film structure corresponds to the Pd seed (grey), Co/Pd underlayer (pink), Co dot (blue), Ta cap (green) and air (white). The calculated reflectivity from these depth profiles are shown as solid lines in (**b**). The Born-simulated reflectometry patterns from the OOMMF simulations are shown as dashed lines in (**b**) and (**c**). Error bars in *q*_z_ identify machine precision; error bars in normalized reflectivity are defined by the s.d. and scale with the square root of the number of measurements.

To demonstrate the SL, we have used polarized neutron reflectometry (PNR) to confirm that the chiral texture in the vortex-state Co dots is imprinted into the Co/Pd underlayer, as PNR is sensitive to structural and magnetic depth profiles of thin films and multilayers^39^,^40^. The hybrid structure was first prepared into the SL state at remanence. By using a position-sensitive area detector, both specular and off-specular scattering (unpolarized) were first captured, as shown in Fig. 4a. PNR measurements were then performed with a perpendicular guide field of 6 mT, placing in-plane magnetic contrast in the spin-flip channel and nuclear contrast in the non-spin-flip channel, as discussed in Methods. Precise scans along *q*_*z*_ isolate the specular reflection and off-specular scattering from the first-order rod, which is parallel to the specular reflection, shown as symbols in Fig. 4b,c, respectively. The specular reflection (*q*_*x*_=0) identifies strictly the nuclear and magnetic depth profile of the hybrid structure while the off-specular reflection, shown at *q*_*x*_=3.6 μm^−1^, includes in-plane contributions from the periodic dot and SL.

The specular reflectivity shows clear oscillations in both the spin-flip and non-spin-flip channels. Since the coherence of the neutron beam in the transverse direction is smaller than the dot diameter, the model used to fit the data was an incoherent sum of scattering from different regions of the dot array. The fitted nuclear and magnetic depth profiles (*χ*^2^=0.70) are shown in Fig. 4d, and the calculated scattering is shown in Fig. 4b as solid lines. The nuclear depth profile matches the designed structure very well. Depth profile of the in-plane magnetization captures not only the entire thickness of the Co dot but also extends into the Co/Pd underlayer by 3 nm (solid red curve), indicating an imprinted interfacial layer. The thickness of the imprinted layer is consistent with thickness of the ion-irradiated region estimated from simulations (Supplementary Fig. 2)^41^. It is this layer that separates the singularities at the top and bottom interfaces and provides the topological protection of the skyrmions, as shown by the magnetization measurements in Fig. 3. The PNR-measured in-plane moment in the interfacial layer is slightly larger than that of the Co dot, likely resulting from the local lateral exchange coupling of the irradiated Co/Pd region to the surrounding unirradiated Co/Pd. Since specular PNR is insensitive to perpendicular magnetization, no net in-plane magnetization is detected from PNR underneath the imprinted interfacial layer or in Co/Pd regions between the irradiated dots, consistent with the PMA in unirradiated Co/Pd.

As confirmation of the model fit for the PNR, the off-specular reflectivity was also simulated using the depth profile from the specular fit. Specifically, a model was constructed in the object-oriented micromagnetic framework (OOMMF) simulation platform (Supplementary Fig. 3) with a structural and magnetic depth profile matching that obtained from the specular measurement. Using a plane-wave Born-approximation model, the reflectometry pattern was then simulated for both the specular and off-specular reflections. Born-OOMMF simulations are shown as dashed lines in Fig. 4b,c. Unfortunately, Born approximation simulations cannot capture dynamic scattering at low *q* such as the critical edge (Fig. 4b; *q*_*z*_<0.1 nm^−1^) in the specular reflectivity or refraction effects when the incident or scattered beam is parallel to the sample surface. Our model results, however, show a clear consistency with the experimental data in the period and relative height of the oscillations. Since the off-specular reflection is sensitive to the in-plane magnetic and structural details, the ability to directly reconstruct these details using a feedback-type approach is strong evidence for the accuracy of the model.

PNR data were also collected for an in-plane guide field (not shown). In this geometry, components of the in-plane magnetization parallel and perpendicular to the field scatter separately into the non-spin-flip and spin-flip channels, respectively; the fit again converged for the same nuclear and magnetic structure shown in Fig. 4d, giving further verification of the depth-dependent model. The neutron scattering presents the crucial piece of evidence that artificial skyrmions are indeed imprinted by the vortex-state Co dot into the Co/Pd underlayer. For samples without the irradiated underlayers, it is likely that the imprinting does not extend as far (or is absent altogether). The smooth variation of the magnetization through the interface between the dot and underlayer reveals that the imprinted structures reflect the chiral structure from the vortex dots, which have been shown to have chiral and polar ordering. Traversing across the centre of each Co/Pd dot structure at the imprinted interface constitutes a 360° rotation of the spin orientation, corresponding to a skyrmion number of *Q*=1, as shown schematically in Fig. 3 for the SL. The imprinted chiral structure over the entire sample is indeed a Bloch SL.

### Magnetoresistance

**Figure 5:**
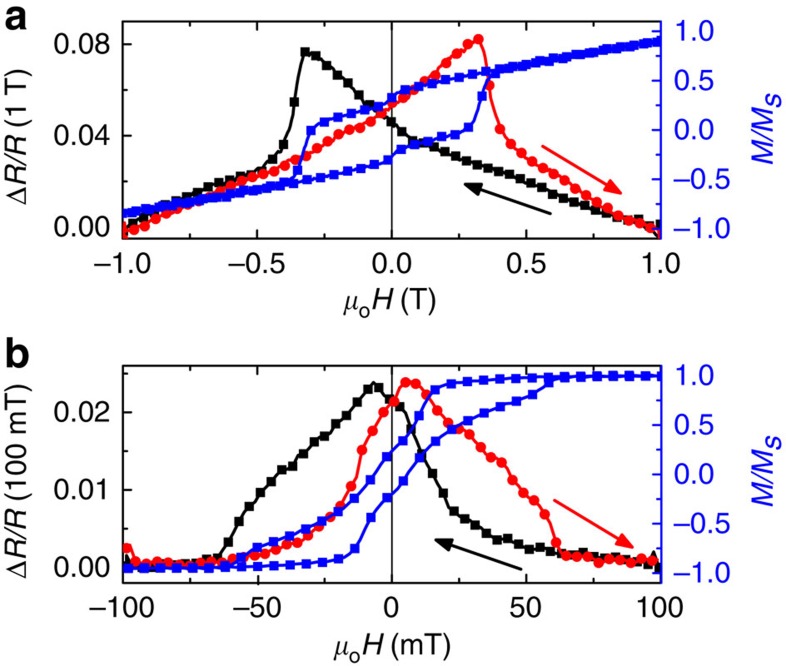
Anisotropic magnetoresistance (AMR). (**a**) Perpendicular AMR, and (**b**) transverse AMR results (black and red curves for descending and ascending field sweeps, respectively), superimposed with (**a**) perpendicular hysteresis loop of the Co/Pd and (**b**) in-plane loop of the Co dots (blue curves), respectively, showing the AMR sensitivity to the imprinted spin texture in the Co/Pd underlayer.

It has been previously shown that, due to their complex spin configurations, magneto-transport behaviour in skyrmion materials presents particular opportunities^12^,^13^,^42^,^43^. Magneto-transport measurements are shown in Fig. 5, showing (panel a) perpendicular and (panel b) transverse anisotropic magnetoresistance (AMR). The perpendicular AMR shows an expected magnetic field dependence, with the AMR peak positions coinciding with the coercive fields of the Co/Pd underlayer perpendicular loop (Fig. 5a) and R(*θ*)=*R*_*T*_+Δ*R* cos^2^*θ*, where resistance *R* depends on the angle *θ* between the applied field and current direction, and *R*_*T*_ is the resistance when *θ*=90°. The signal is small because much of the current is shunted through the Pd seed layer. With the field applied in-plane but perpendicular to the current, the transverse AMR shows a sensitivity to the vortex nucleation and annihilation events. At large fields, the dots are in the single domain configuration, with their moment perpendicular to the current path. On nucleation the vortex forms a flux-closure structure, which can be approximated as four orthogonally configured magnetic regions, thus roughly half of the vortex remains perpendicular to the current and half becomes parallel, and the latter contributes to a larger resistance *R*_//_=*R*_*T*_+Δ*R* as *θ*=0. The transverse AMR peak positions coincide with the coercivities of the in-plane loop of the Co dots (Fig. 5b) where the parallel components are at the maximum. The discrete nature of the Co dots suggests that the AMR signal should come from spin textures in the Co/Pd underlayer. The correlation between the transverse AMR and the in-plane vortex structure of the Co dots indicates a strong coupling between the dots and the underlayer, which is further evidence of the imprinted SL.

## Discussion

We have successfully achieved room-temperature artificial SLs in the ground state over extended areas, thus defining a platform for exploring the properties and behaviours as well as the use of SL in novel technological concepts. The system is constructed by fabricating circularity controlled Co nanodots on a Co/Pd underlayer with PMA. Circularity control is imposed by the fabrication of asymmetric dots and confirmed by magnetometry and direct magnetic imaging. Polarity control is realized by the application of a perpendicular magnetic field during the vortex nucleation to achieve SL, VL and ML, as confirmed by remanent magnetization and topology-dependent magnetization reversal of the underlayer. The vortex structure in the Co dots is imprinted into the Co/Pd underlayer through reduction of the interfacial PMA via ion irradiation. This is a critical step that allowed the realization of the SL at the interfacial region of the underlayer, with imprinted spin textures embedded in the matrix of unirradiated Co/Pd. The imprinted SL in the Co/Pd is directly confirmed by PNR, and is also manifested in the AMR measurements and micromagnetic simulations. These artificially constructed SLs are stable at room temperature and in the absence of magnetic field, controlled by the exchange interaction and magnetostatic energies, in contrast to the skyrmion phase in conventional systems, which tend to exist in limited temperature-magnetic field parameter space^3^,^6^,^7^,^14^,^15^,^16^,^17^. These foundational results present a new path in skyrmion research on the mesoscale, at and above room temperature.

## Methods

### Sample fabrication

Samples were prepared by a three-step process. In step one, PMA thin films with a nominal structure of [Co (0.5 nm)/Pd (1 nm)]_10_ were grown on naturally oxidized Si substrates with a Pd seed layer by DC magnetron sputtering in a 0.67-Pa Ar atmosphere (base pressure ∼1.2 × 10^−6^ Pa). In the second step, following a standard e-beam lithography procedure, hexagonal arrays of edge-cut asymmetric holes with diameter of 560 nm and centre-to-centre spacing of 1 μm were patterned into a ∼400-nm thick PMMA polymer layer, which was spin-coated onto the Co/Pd film. After development, the exposed Co/Pd was irradiated by 1 keV Ar^+^ plasma (with a fluence of 200 mA cm^−2^) for 3 s. Using the stopping range in matter (SRIM) simulation platform^41^, calculations suggest damage to the multilayer structure up to a 4-nm depth (Supplementary Fig. 2; Supplementary Note 2); the PMMA sufficiently protects the undeveloped regions. During the third step, 32 nm thick asymmetric Co dots were grown over the irradiated regions following a standard lift-off procedure, as illustrated in Figs 1a and 2. The entire patterned area was 1 cm^2^. Layer thicknesses were confirmed from witness samples made at the same time and measured by X-ray reflectivity; lateral dimensions were confirmed by scanning electron microscopy.

### Setting the skyrmion state

Once the samples were fabricated, an appropriate magnetic field sequence was applied at room temperature to prepare the skyrmion state. First, the Co/Pd underlayer was saturated in the positive OOP direction (*μ*_o_H_OOP_=1.5 T). Then, a static field was applied in the negative OOP direction (*μ*_o_H_OOP_=−100 mT), which was much weaker than the reversal field of the underlayer and the Co/Pd remained positively saturated. A moderate in-plane field was then applied parallel to the flat edge of the asymmetric Co dots, driving them to a saturated state (*μ*_o_*H*_OOP_=−100 mT, *μ*_o_*H*_IP_=100 mT). Removing this field facilitated the nucleation of a vortex in each of the dots with a well-defined circularity^26^,^44^; the static perpendicular field biased the vortex core polarity at the nucleation event to be opposite to the Co/Pd underlayer; by irradiating the Co/Pd region underneath the Co vortex dots, the competition between the underlayer's PMA and the dot's magnetostatic energy was circumvented, allowing imprinting of the vortex structure and ensuring that the underlayer orientation does not influence the core polarity. For comparison, a VL was formed by applying the static perpendicular field parallel to the underlayer. Finally, an ML was prepared in the absence of a guide field and is expected to have a random polarity distribution.

### Characterizations

Characterizations were all done at room temperature. Magnetometry was measured using a vibrating sample magnetometer in both the perpendicular and in-plane geometries. In the in-plane case the field was applied parallel to the flat edge of the dots. FORCs were measured following prior procedures^35^,^36^,^37^ where the sample was first positively saturated, then brought to progressively more negative reversal fields, and the magnetization was measured under increasing applied field back to positive saturation. Magnetic imaging was performed using SEMPA at the Center for Nanoscale Science and Technology at NIST, and MTXM^29^ at the Advanced Light Source. MFM was also performed. Magneto-transport measurements were performed using a pulsed bipolar current supply (±5 mA), measured on a nanovoltmeter.

Polarized neutron reflectivity was performed at the NIST Center for Neutron Research on the MAGIK reflectometer with *λ*=0.5 nm neutrons. Measurements are presented as a function of wave vector transfer magnitude, *q*_*x*, *y*, *z*_, with *q*_*z*_ indicating the OOP direction. The spin polarization of the neutrons was selected to be up (+) or down (−) before and after scattering from the sample using supermirror polarizers and Al-coil spin flippers, allowing measurement of the spin-flip (R_incident scattered_: R_+−_ and R_−+_) and non-spin-flip (R_++_ and R_−−_) signals. A perpendicular guide field of 6 mT was used to set the neutron spin direction perpendicular to the film plane. For specular measurements, with a perpendicular guide field, the magnetic and nuclear scattering are separated into the spin-flip and non-spin-flip channels, respectively. The spin-flip scattering originates from the component of the magnetization perpendicular to the neutron spin. It is only sensitive to the in-plane magnetic moments and thus originates from the dot and imprinted structures. The specular reflectometry was fitted using the Refl1d software package^45^. The Refl1d calculation by default uses a coherent summation scheme for modelling the depth-dependent scattering length density—meaning the neutron wave function is assumed to be laterally larger than the features and thus the scattering is treated as the in-plane average. In this work the features of the sample are comparable to the neutron's lateral distribution, and thus the net signal is a weighted sum of several models corresponding to different regions of the sample—referred to as an incoherent sum (Supplementary Figs 4 and 5; Supplementary Note 3).

### Simulations

Simulations of the irradiation penetration were performed using the SRIM simulation platform^41^ modelling 1 keV Ar^+^ normally incident on a film with the same structure as the experimental underlayer. The results suggest that irradiation damages the top three bilayers, corresponding to ∼4 nm. OOMMF simulations were used to model the magnetic structure of the hybrid structure. Simulations were performed using the bulk values for the saturation magnetization (*M*_*S*_ (Co)=1.40 × 10^6^ A m^−3^, *M*_*S*_ (unirradiated Co/Pd, irradiated CoPd)=3.02 × 10^5^ A m^−3^), magneto-crystalline anisotropy (*K*_*U*_ (Co, irradiated CoPd)=0 J m^−3^, *K*_*U*_ (unirradiated Co/Pd)=8 × 10^5^ J m^−3^) and exchange stiffness (*A* (Co)=3.0 × 10^−11^ J m^−1^, *A* (unirradiated Co/Pd and irradiated CoPd)=5 × 10^−12^ J m^−1^) with a rectangular mesh of 4 × 4 × 2 nm; the irradiated region was treated as an embedded uniform cylinder located under the dot with a thickness of 4 nm, consistent with the SRIM simulations and PNR results, and having the same saturation magnetization and exchange stiffness as the unirradiated underlayer, but with *K*_*U*_=0. Simulations show that with the polarity setting field, the chiral structure and core structure are imprinted into the underlayer; in contrast, without the conditioning field the hybrid structure is not in the skyrmion state (Supplementary Fig. 3).

## Additional information

**How to cite this article:** Gilbert, D. A. *et al*. Realization of ground-state artificial skyrmion lattices at room temperature. *Nat. Commun.* 6:8462 doi: 10.1038/ncomms9462 (2015).

## Supplementary Material

Supplementary Information Supplementary Figures 1-5, Supplementary Notes 1-3 and Supplementary Reference
