# Supplementary material for: Realization of ground-state artificial skyrmion lattices at room temperature
Source: Nat Commun. 2015 Oct 8;6:8462. doi: 10.1038/ncomms9462 (PMC4633628; doi:10.1038/ncomms9462)
Supplement: Supplementary Information — Supplementary Figures 1-5, Supplementary Notes 1-3 and Supplementary Reference [file ncomms9462-s1.pdf]

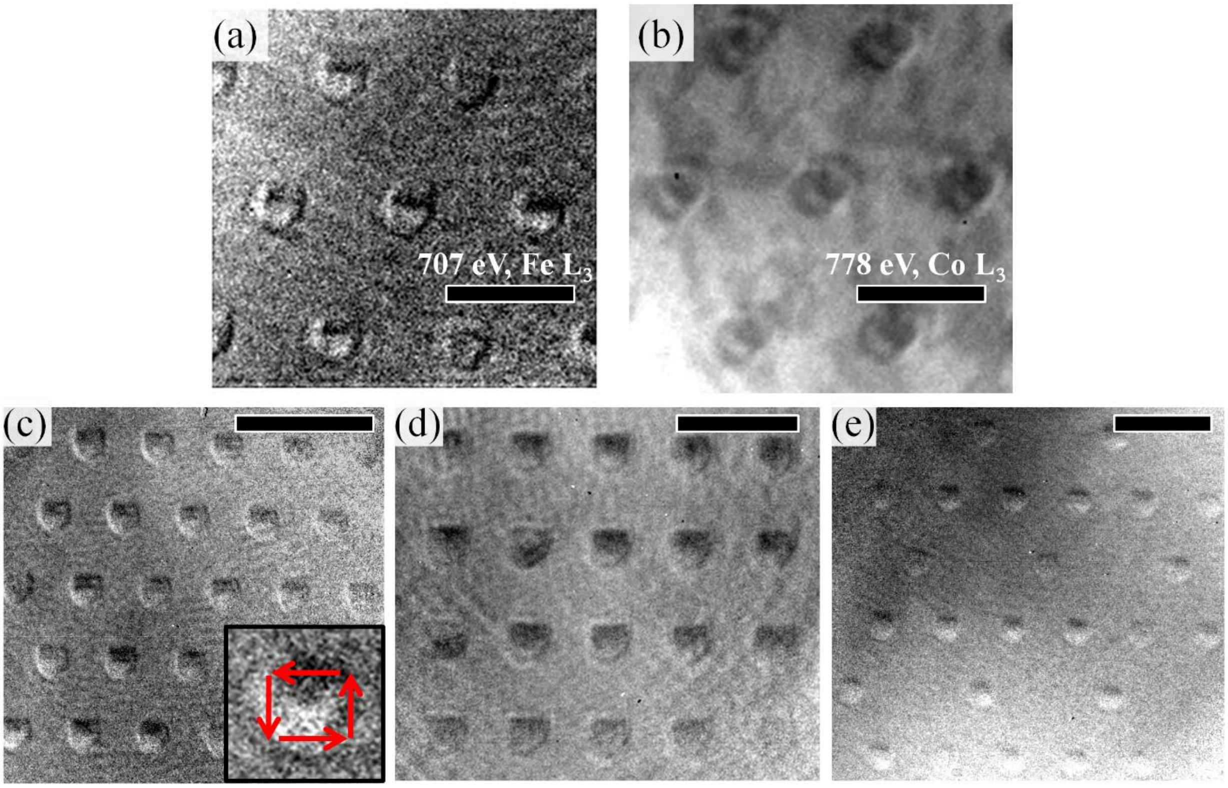

Supplementary Figure 1. Magnetic transmission x-ray microscopy. (Top row) MTXM images of 385 nm diameter Py dots on as-grown Co/Pd multilayer films with PMA. Images are taken at the (a) Fe-L_3 edge (707 eV) and (b) Co L_3 edge (778 eV). (Bottom row) MTXM images taken at the Co L_3 edge (778 eV) of Co dots on as-grown Co/Pd multilayer films (c) 470 nm diameter in hexagonal arrays, (d) 490 nm diameter in square arrays, and (e) 410 nm diameter in honeycomb arrays. Scale bar is 1 μm in panels (a, b, d) and 2 μm in (c, e).

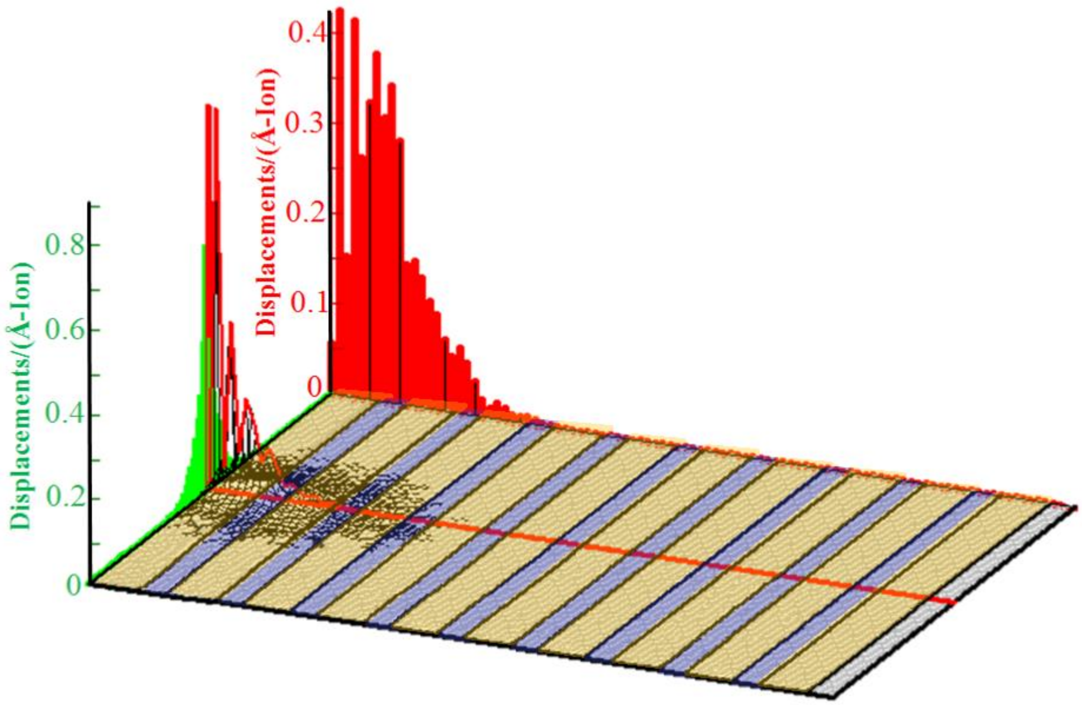

Supplementary Figure 2. SRIM model of irradiation damage. A penetration of the top 3 bilayers of the underlayer (~4nm depth) is shown. The horizontal plane represents the cross-sectional view of the Co (grey) / Pd (yellow) multilayer.

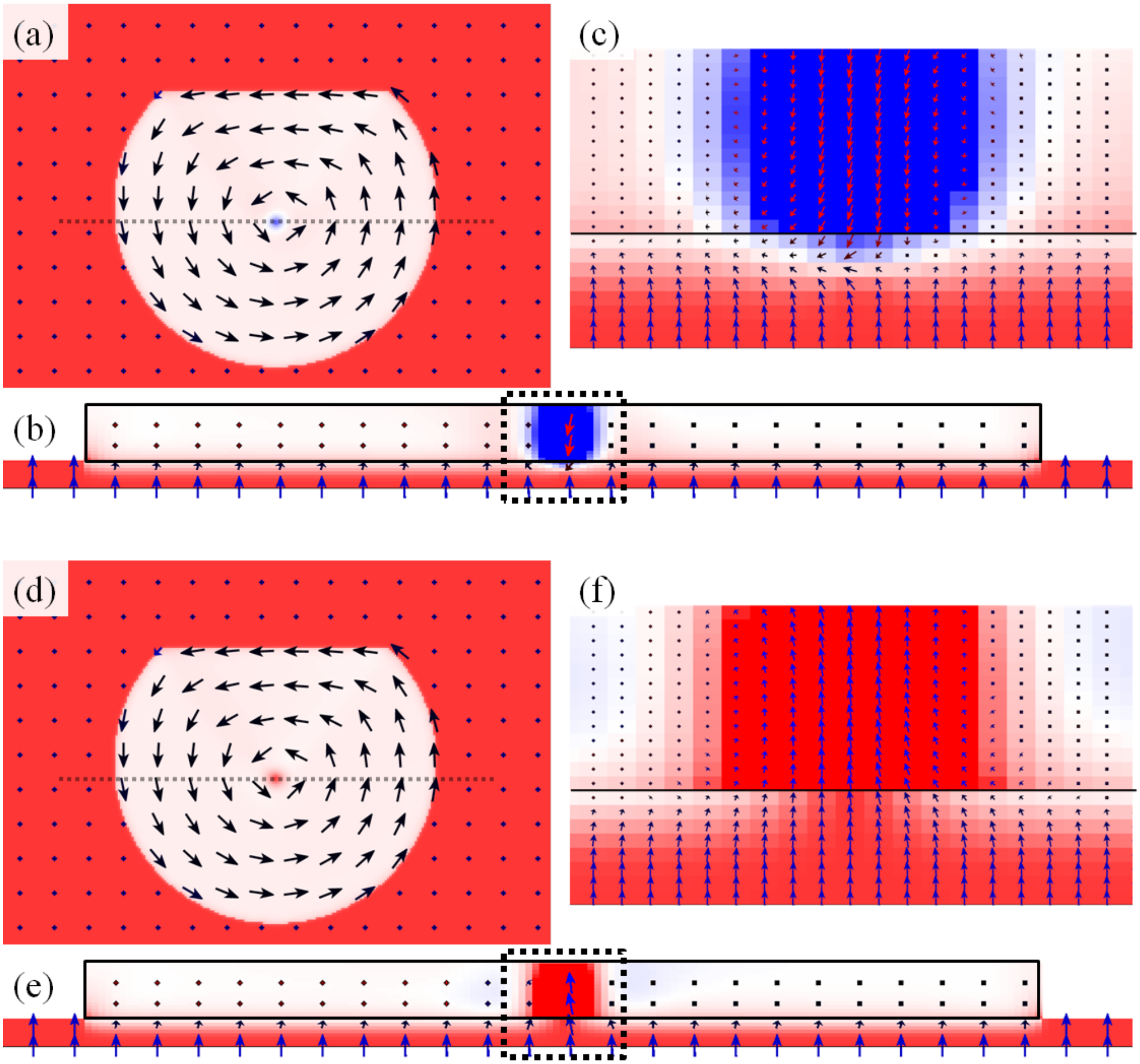

Supplementary Figure 3. Micromagnetic simulations. OOMMF simulations of hybrid structure at remanence after field processing (a-c) with and (d-f) without a polarity setting field. The top view (a, d) shows the imprinted chiral structure, while the cross-section view, taken at the dashed line in (a, c) is shown in (b, e). In the side-view the solid line indicates the dot structure; the dashed box indicates the zoomed-in region, shown in (c, f); the zoomed-in region shows imprinting of the core only in the irradiated region.

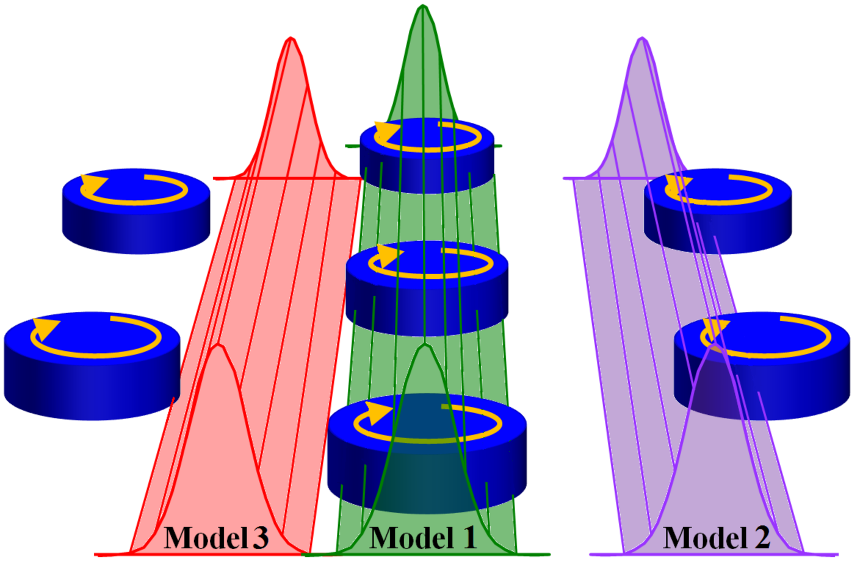

Supplementary Figure 4. Schematic diagram of neutron model pieces. Model piece 1, 2 and 3 represents a neutron which traverses over the center of many dots, part of the dots, and un-patterned areas of the film, respectively.

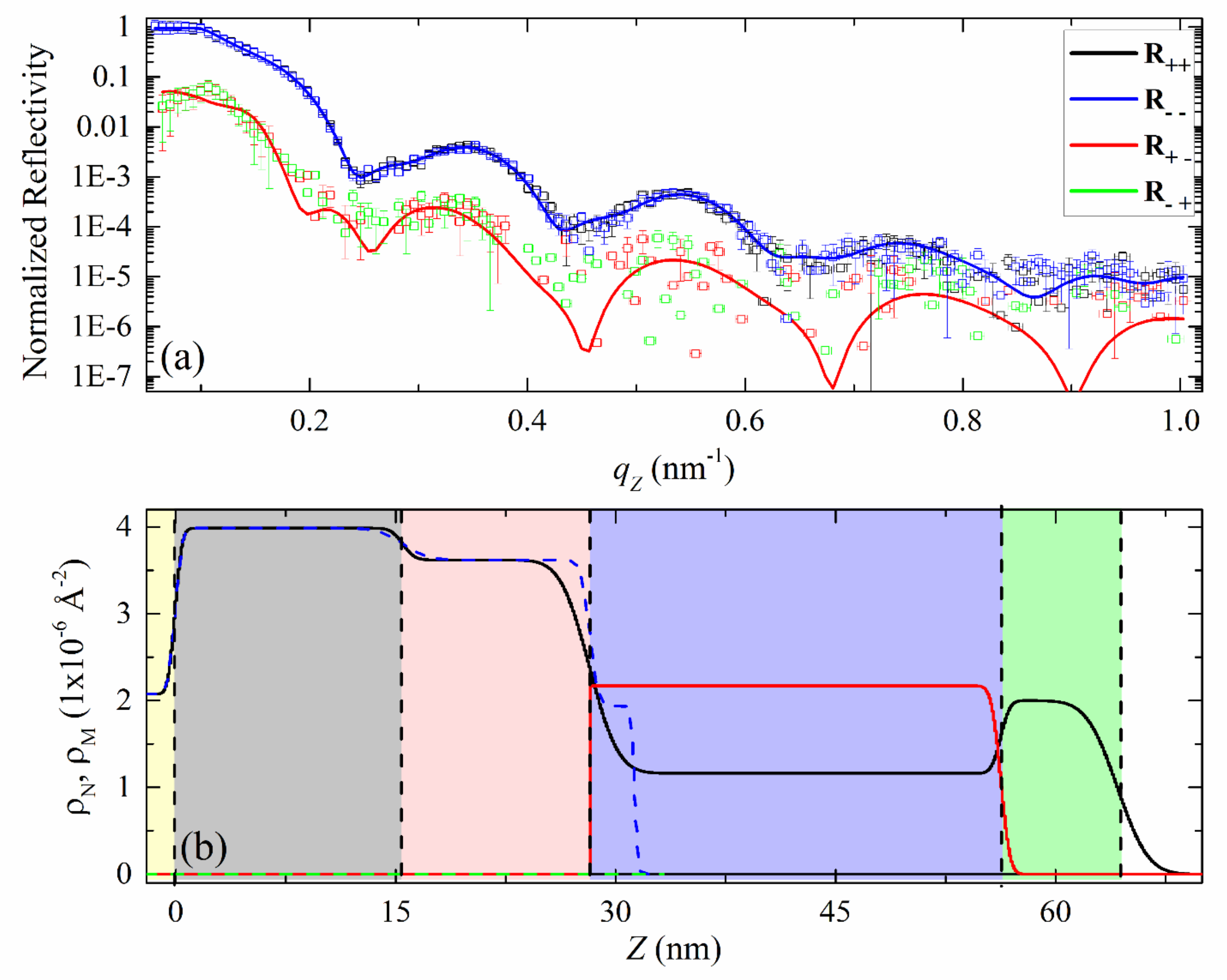

Supplementary Figure 5. Polarized neutron reflectometry. (a) A fitted neutron reflectivity pattern (curves), restricted to not having an imprinted structure, shows poor agreement with experimentally measured data (symbols). The corresponding depth-dependent nuclear (ρ_N, solid black and dashed blue curves) and magnetic (ρ_M, solid red and dashed green curves) scattering densities are shown in (b), over the protected film (dashed curves) and the vortex region (solid curves). On top of the Si substrate (yellow region), at increasing depth, the film structure corresponds to the Pd seed (grey), Co/Pd underlayer (pink), Co dot (blue), Ta cap (green), and air (white).

Supplementary Note 1

Samples with NiFe (Py) dots on unirradiated Co/Pd that rely on the interfacial exchange interaction to imprint the skyrmion structure were fabricated following the procedure outlined in the main text. Magnetic transmission x-ray microscopy (MTXM) images were taken using the XM-1 microscope at the Advanced Light Source in Berkeley, CA, on samples grown on SiN membrane. Images of the in-plane magnetic structure are shown in Supplementary Fig. 1 recorded at the (a) Fe L_3 edge (707 eV) and (b) Co L_3 edge (778 eV). The images confirm the circularity control in the Py dots (probed by the Fe component); however, no chiral structure was observed at the Co edge. This is in agreement with OOMMF simulations indicating that the imprinting of a skyrmion structure is located only in a very thin interfacial layer, which seems to provide insufficient magnetic contrast with MTXM.

MTXM images were also taken of Co dots patterned onto unirradiated Co/Pd underlayers, similar to the system in the main text, but grown on a SiN membrane. These images show circularity control in a variety of asymmetric dots patterned into arrays of (c) hexagonal, (d) square, and (e) honeycomb lattices. However, it was not possible to distinguish the dot and any imprinted structure.

Supplementary Note 2

The SRIM results, shown in Supplementary Fig. 2, suggest that irradiation damages the top 3 bilayers, corresponding to ~4 nm. OOMMF simulations followed the field conditioning presented in the main text, with [Supplementary Fig. 3 (a-c)] and without the polarity setting field [Supplementary Fig. 3(d-f)]. The top-view, panels (a, d), shows the imprinting of the chiral

structure into the irradiated region. The cross-section view (b, e), along with zoomed-in view (c, f), shows that the chiral structure and core structure are imprinted only superficially in the underlayer. The model without the conditioning field is shown to have a polarity parallel to the underlayer, and thus is not a skyrmion structure, as its skyrmion number would be 0.

Born approximation simulation for the neutron reflectivity used the remanent state OOMMF model of the skyrmion configuration as input for the magnetization and demagnetization-field configuration, and an identical geometry for the nuclear scattering. These three models were combined to calculate the neutron scattering using a plane-wave approximation. The neutron was assumed to have a coherence length along the neutron path of travel much longer than the OOMMF simulated volume and a smaller Gaussian-distributed lateral width of 500 nm along the transverse beam direction.[1] Accordingly, the overlap of the neutron wave function with the dot and skyrmion will depend on the spatial location of the neutron. Each neutron sees a different potential which results in different scattering. The measured signal is thus an incoherent (e.g. arithmetic) sum of all the scattered neutrons. The neutron flux is expected to be spatially uniform, and the incoherent sum is calculated by stepping the wave function across the simulated volume in 100 nm increments (approximately 10 overlapping bands). Within a band the scattering potential is calculated as a weighted sum, reflecting the distribution of the neutron wave function envelope. The scattering from each region was then added. This approach was shown to accurately reproduce the major features of the experimental data, shown in the main text Figs. 4(b) and 4(c). A similar broad-binning approach is used in the Refl1d model, discussed in the main text and below.

Supplementary Note 3

The neutron measurements were performed with a perpendicular guide field, making the neutron sensitive to any net in-plane magnetization within the coherence length of the neutron. The neutron coherence length on the MAGIK reflectometer was measured recently to be 100μm along the direction of the neutron's travel and <1 μm in the transverse direction.[1] The scattering potential seen by the neutron is the arithmetic average of everything within its coherence length. Thus, a single neutron will sample between 100 and 200 skyrmion and vortex structures along its direction of travel. Further consideration of the transverse coherence length reveals that each skyrmion and vortex structure contributes only a fraction of its total scattering potential in this direction based on the neutron wave function overlap. As a result, using a simple model which utilizes a broad coherent addition, as is typically done with Refl1d, will not work. This same problem is discussed above for the Born simulations and was resolved using a binning approach. Following a similar approach the data were fitted using a three piece model, as shown schematically in Supplementary Fig. 4: piece 1 represents a neutron which traverses over the center of many dots and thus scatters with the nuclear structure of the dots on the film, but due to the chiral nature of the structure has no magnetic scattering; piece 2 represents a neutron which traverses part of the dots, thus (for the ordered chiral state) experiences scattering from an accumulated net magnetic moments; piece 3 represents un-patterned areas of the film, where either there were no patterned dots or the neutron coherence does not overlap with many dots. The nuclear structure in the three models was all restricted to be the same along their thickness but with different densities, while the magnetic structure was allowed to vary.

The complex structure of the model means that several combinations of parameters could provide reasonable fits for these data sets. As a result it is not possible to determine a unique

solution for the imprinting. However, the imprinted structure is consistent amongst all of the converged models. Further, the accuracy of the off-specular Born simulation strongly supports the correctness of the model.

Our best fit model which does not have an imprinted structure is shown in Supplementary Fig. 5. This profile was allowed to vary the nuclear profile, but restricted from having an imprinted structure; the entire magnetic signature comes from the Co dot (solid red curve). The resultant modeled reflectivity pattern shown in (a) has some significant deviations from the experimental data (shown on a log scale) in the spin-flip channel: the calculated reflectivity at low q_z increases approaching q_z = 0, while the experimental data decrease; the double-bump feature at q_z = 0.22 nm^{-1} does not appear in the experimental data; the minimum at q_z = 0.45 nm^{-1} is displaced along q_z relative to the data; and the magnitude of the second oscillation in the reflectivity is reduced relative to the data. Deviation in the minimum at q_z = 0.45 nm^{-1} is particularly concerning because the oscillation period is a direct measure of the thickness profile. It can be shown directly from the Bragg condition that thinner layers contribute to longer oscillation frequencies. In Supplementary Fig. 5(a) the simulated oscillation period is longer than the oscillation period in the experimental data, indicating that the magnetic structure in the model is too thin, and motivating the inclusion of the imprinted region.

Supplementary Reference

1 Majkrzak, C. F. *et al.* Determination of the effective transverse coherence of the neutron wave packet as employed in reflectivity investigations of condensed-matter structures. *Phys. Rev. A* **89**, 033851 (2014).
